# Comparison of the analyses of the XV^th ^QTLMAS common dataset II: QTL analysis

**DOI:** 10.1186/1753-6561-6-S2-S2

**Published:** 2012-05-21

**Authors:** Olivier Demeure, Olivier Filangi, Jean-Michel Elsen, Pascale Le Roy

**Affiliations:** 1 INRA, UMR1348 PEGASE, Domaine de la Prise, 35590 Saint-Gilles, France; 2 Agrocampus OUEST, UMR1348 PEGASE, 65 rue de St Brieuc, 35042 Rennes, France; 3 INRA, UR0631 SAGA, Chemin de Borde Rouge, BP 52627, 31326 Castanet-Tolosan, France

## Abstract

**Background:**

The QTLMAS XV^th ^dataset consisted of the pedigrees, marker genotypes and quantitative trait performances of 2,000 phenotyped animals with a half-sib family structure. The trait was regulated by 8 QTL which display additive, imprinting or epistatic effects. This paper aims at comparing the QTL mapping results obtained by six participants of the workshop.

**Methods:**

Different regression, GBLUP, LASSO and Bayesian methods were applied for QTL detection. The results of these methods are compared based on the number of correctly mapped QTL, the number of false positives, the accuracy of the QTL location and the estimation of the QTL effect.

**Results:**

All the simulated QTL, except the interacting QTL on Chr5, were identified by the participants. Depending on the method, 3 to 7 out of the 8 QTL were identified. The distance to the real location and the accuracy of the QTL effect varied to a large extent depending on the methods and complexity of the simulated QTL.

**Conclusions:**

While all methods were fairly efficient in detecting QTL with additive effects, it was clear that for non-additive situations, such as parent-of-origin effects or interactions, the BayesC method gave the best results by detecting 7 out of the 8 simulated QTL, with only two false positives and a good precision (less than 1 cM away on average). Indeed, if LASSO could detect QTL even in complex situations, it was associated with too many false positive results to allow for efficient GWAS. GENMIX, a method based on the phylogenies of local haplotypes, also appeared as a promising approach, which however showed a few more false positives when compared with the BayesC method.

## Background

In the past years, the availability of large sets of genetic markers has allowed the implementation of genome-wide association studies (GWAS) in livestock. Many methods have been developed for GWAS, most of them hypothesizing an additive QTL effects. However, more complex situations exist, with dominance, interactions between genes (epistasis) or parent-of-origin effects (imprinting) [1,2]. The XV^th ^QTLMAS dataset was simulated for a single quantitative trait controlled by 8 QTL with additive, epistatic or imprinting effects. Comparing the results obtained by the different groups should provide insight into determining which method is best fitted for each complex case. In addition, until now, most of the GWAS studies have been performed in ruminant species (large number of progeny per sire, only one or two per dam). In order to establish whether this kind of approach is also adapted to pig and chicken designs, this dataset was designed for medium-sized full sib families.

## Methods

### Simulated data

The simulated data set was described by Elsen et al. [3]. Briefly, the population comprised 3,000 individuals born from 20 sires and 200 dams. Within each family, 10 progenies were assigned phenotypes and marker genotypes. A total of 10,000 SNPs carried by 5 chromosomes of 1 Morgan each were simulated. Eight QTL were simulated: one quadri-allelic additive QTL with a large effect on Chr1, two linked QTL in phase on Chr2, two linked QTL in repulsion on Chr3, one imprinted QTL on Chr4 and two interacting QTL on Chr5. Random noise was added, giving an heritability coefficient of 0.30. The marker density, linkage disequilibrium (LD) and minor allele frequency (MAF) were similar to real life parameters.

### Methods used by the participants

The methods used were either genomic, considering all SNPs in a single analysis, or local, testing SNPs one by one (Table 1).

**Table 1:** Methods and models used by the participants at the XV^th ^QTLMAS

Authors	Genomic approaches						Single QTL scan		
	GBLUP	LASSO	Bayes				Regression	Mixed model	
			A	B	C	Cπ		Full	approximated
Dashab et al.					x			3 versions	
Fu et al.									EMMAX type
Nadaf et al.	x		x	x			x		GRAMMAR
Schurink et al.						x			
Usai et al.		x							
Zeng et al.	x			x		x			

In the genomic group, the GBLUP method [4,5] assumed that all SNPs may contribute to trait variability, while all other methods considered the SNP population as a mixture of a small number of SNPs involved in this variability and a large number of neutral SNPs. This mixture situation was solved by different LASSO approaches (the classical LASSO used by Nadaf et al. [4] was compared to two new strategies used by Usai et al. [6]) and by MCMC Bayes techniques: Bayes A [4], Bayes B [4,5], Bayes C [7] and Bayes Cπ [5,8].

Various methods scanning successive candidate gene locations were proposed. Nadaf et al. [4] made use of the half sib regression technique described by Knott et al. [9], while all other methods were based on a mixed model in which a random polygenic effect was added to the fixed QTL effect. Dashab et al. [7] compared different ways of processing this marker information: single marker analysis, phasing of genotypes and haplotype analysis, and clustering of haplotypes based on local genealogies using the GENMIX model of Sahana et al. [10]. Two approximations of the full mixed model were tested by Nadaf et al. (the GRAMMAR method described by Aulchenko et al. [11] and an EMMAX-type approach described by Zhang et al. [12]), in which the polygenic variance was estimated before scanning for QTL.

### Comparison of the results

Results from the five groups were compared based on four criteria: i) the number of true QTL detected (*i.e*. a QTL mapped at less than 5 cM from a simulated QTL); ii) the number of false positive QTL (*i.e*. the distance to the closest true QTL exceeded 5 cM); iii) the accuracy of the QTL location (*i.e*.the distance between the estimated QTL location and the true location); iv) the accuracy of the QTL effect estimation.

Since the results of Nadaf et al. [4] were only presented in a graphical way, no numerical indication will be provided for their methods.

## Results

A global view of the performances of the different methods is given in Table 2. The estimated positions of the true QTL on each chromosome, when detected, are assembled in Table 3, and the estimation of the QTL effect is presented in Table 4. On the whole, BayesC, LASSO and GENMIX showed the highest power (and 7 out of the 8 QTL), with a rather high number of false positives for GENMIX and a huge number of false positions with the LASSO.

**Table 2:** Comparison of QTL mapping results

Method	Authors	QTL detected	False positives	Missing QTL (a)	Mean distance to the QTL (cM)
GBLUP	Zeng	5	0	3,5,8	1.32
BayesB	Zeng	6	3	7,8	1.21
BayesC	Dashab	7	2	8	0.96
BayesCπ	Schurink	5	0	5,6,8	0.83
	Zeng	5	1	6,7,8	0.45
LASSO1	Usai	7	Numerous	6	0.83
LASSO2	Usai	7	Numerous	6	0.74
LASSO3	Usai	7	Numerous	6	0.29
MM single SNP	Dashab	5	3	3,6,8	0.73
MM Haplotype	Dashab	5	6	2,6,8	0.61
MM Phylogeny	Dashab	7	5	8	1.07
EMMAX	Fu	3	1	3,5,6,7,8	0.43

(a) 2: Chr2, 81,9cM; 3: Chr2, 93,75cM; 5: Chr3, 15cM; 6: Chr4, 32,2cM; 7: Chr5, 36,3cM; 8:Chr5, 99,2cM.

**Table 3:** Localization of the simulated QTL depending on the method/model used

Chromosome		Chr1	Chr2	Chr2	Chr3	Chr3	Chr4	Chr5	Chr5
True position		2.85	81.9	93.75	5	15	32.2	36.6	99.2
GBLUP	Zeng	2.95	83.0		4.75		28.2	no	no
BayesB	Zeng	2.85	83.0	93.7	4.75	15.8	27.9	no	no
BayesC	Dashab	2.75	83.1	93.4	4.8	14.8	28.3	35.1	no
BayesCπ	Schurink	1.6	83.1	93.4	2.9	(14.8)	(28.3)	35.1	no
BayesCπ	Zeng	2.75	83.0	93.6	4.6	16.6	no	no	no
LASSO1	Usai	2.90	81.9	95.8	4.8	16.1	28.0	35.1	no
LASSO2	Usai	2.90	81.8	94.0	4.8	16.7	34.9	36.8	no
LASSO3	Usai	2.90	81.8	95.8	4.8	15.8	28	36.8	no
MM single SNP	Dashab	3.55	82.0	no	4.8	16.5	no	36.2	no
MM Haplotype	Dashab	2.5	no	96.0	4.8	14.9	no	35.9	no
MM Phylogeny	Dashab	2.7	82.3	95.8	4.8	11.1	31.7	36	no
EMMAX	Fu	2.9	83.1	no	4.8	no	no	no	no

**Table 4:** Comparison of QTL effect estimations

Chromosome		Chr1	Chr2	Chr2	Chr3	Chr3	Chr4	Chr5	Chr5
True effect^1^		12.	2.	2.	2.	2.	4.	1.27^2^	1.27^3^
GBLUP	Zeng	4.39	1.92		2.31		1.18	1.34	
BayesB	Zeng	2.17	0.68	0.62	0.2	0.15	0.13		
BayesC	Dashab								
BayesCπ	Schurink	1.92	1.28	0.84	1.24	0.97			
	Zeng	7.01	0.69	0.66	1.18	0.24			
LASSO1	Usai	2.22	0.71	0.48	1.43	0.15	0.2	0.12	
LASSO2	Usai	2.09	1.39	2.18	1.67	0.38	0.23	1.42	
LASSO3	Usai	2.22	1.01	0.56	1.58	0.29	0.01	0.67	
MM single SNP	Dashab	4.19	2.1		2.71	0.65		1.31	
MM Haplotype	Dashab								
MM Phylogeny	Dashab								
EMMAX	Fu	4.39	2.08	2.46					

^1 ^Apparent difference between homozygous in trait units

^2 ^Only 63.6% of the homozygous individuals, which were also homozygous for the second interacting QTL, expressed the 2 trait unit effect of this 1^st ^QTL

^3^Only 63.6% of the homozygous individuals, which were also homozygous for the first interacting QTL, expressed the 2 trait unit effect of this 2^nd ^QTL

### Large effect additive QTL (Chr1)

**Figure 1:**
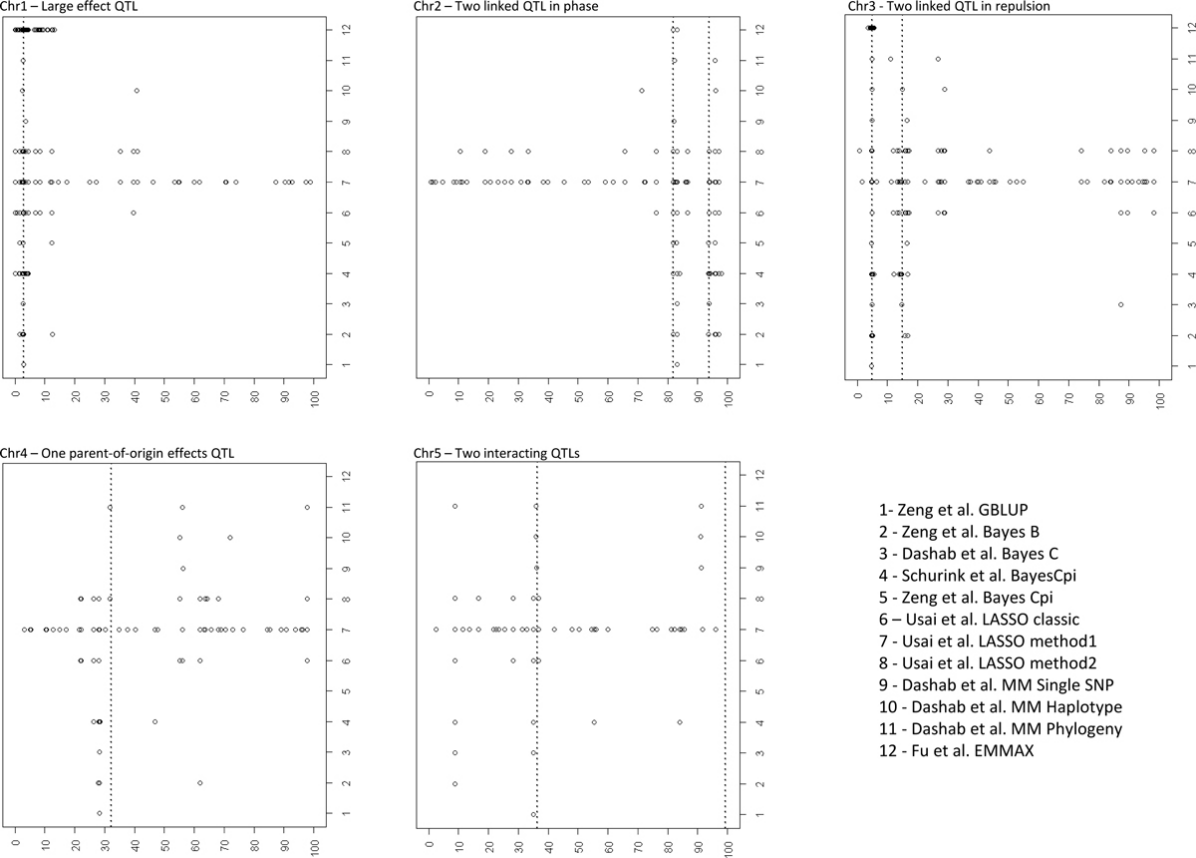
**QTL mapping results for the 12 tested methods**. 1- Zeng et al. GBLUP; 2 - Zeng et al. Bayes B; 3 - Dashab et al. Bayes C; 4 - Schurink et al. BayesCπ; 5 - Zeng et al. Bayes Cπ; 6 - Usai et al. LASSO classic; 7 - Usai et al. LASSO method1; 8 - Usai et al. LASSO method2; 9 - Dashab et al. MM Single SNP; 10 - Dashab et al. MM Haplotype; 11 - Dashab et al. MM Phylogeny; 12 - Fu et al. EMMAX. Dotted lines: simulated QTL locations.

**Figure 2:**
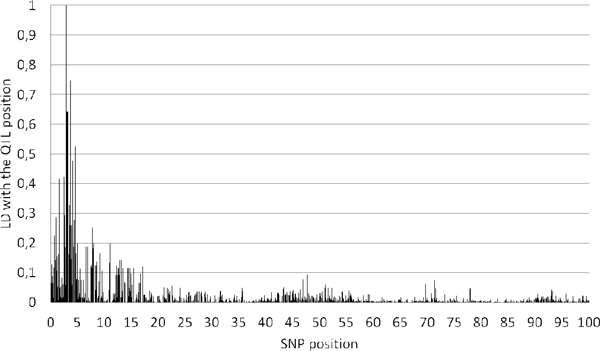
**Linkage Disequilibrium between the simulated QTL (position 2.85 cM) and the chromosome 1 SNPs**.

All groups and methods found this additive QTL with a large effect of 1.28 phenotypic standard deviations (Figure 1A). With the exception of the BayesCπ method used by Shurink et al. [8], the genomic methods gave an estimated location which was very close to the exact one (less than 0.1 cM away) (Table 3). Single SNP analyses were less consistent: the single SNP mixed model used by Dashab et al. [7] positioned the QTL at 0.7 cM from the true QTL, while the same model, approximated with an EMMAX type approach by Fu et al. [13] gave a very precise position (0.1 cM). Adding marker information to the analysis increased location accuracy (haplotype and phylogenybased approaches of Dashab et al. [7]), with a very satisfying performance of the GENMIX method. Surprisingly, two other regions were also often identified at 12.5cM (BayesB and BayesCπ[5], LASSO [6] and the EMMAX-type mixed model [13]) and 40 cM (LASSO [6] and haplotype regression [7]). Local linkage disequilibrium between SNPs around these positions and the QTL may contribute to the occurrence of these false discoveries (Figure 2). However, it is interesting to mention that no false positives occurred with the GBLUP methods. All methods underestimated the variance explained by the QTL, the closest method being the BayesCπ used by Zeng et al. (Table 4).

### Linked QTL in phase (Chr2)

**Figure 3:**
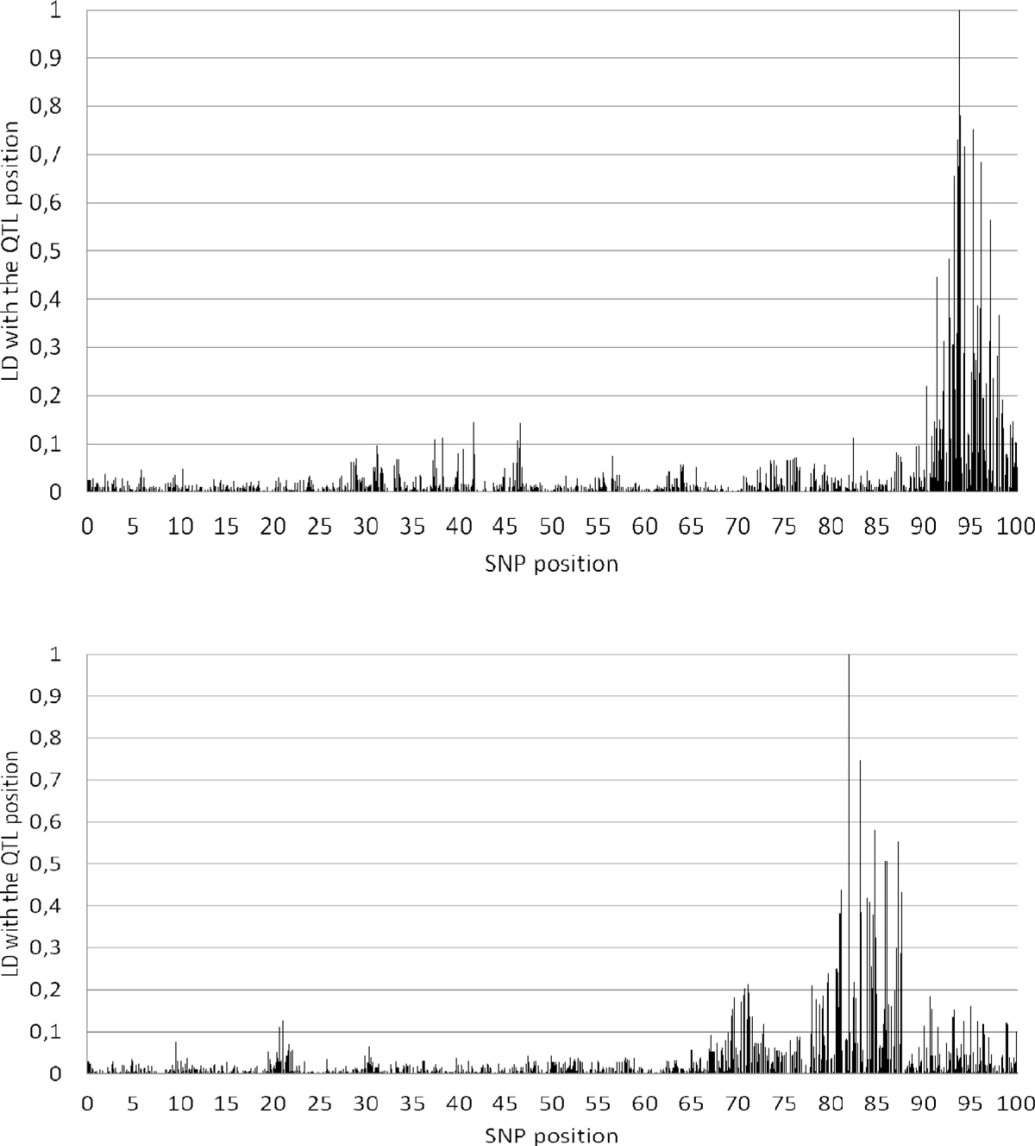
**Linkage Disequilibrium between the simulated QTL (positions 81.9 cM (A) and 93.75 cM (B)) and the chromosome 2 SNPs**.

The two QTL located at 81.9 and 93.8 cM were identified both by the single SNP mixed model based on phylogenies [7] and by all mixture models solved by LASSO or Bayesian strategies. None of these approaches prevailed: the Bayesian methods were very precise for the second QTL, but the other techniques worked fine for the first one (Figure 1B). This first QTL was not identified by the Dashab et al. haplotype regression strategy [7] while the GBLUP [5] and the single marker mixed models, full [7] or approached [13], were unable to detect the second QTL. For these QTL, the single SNP regression methods [7,13] provided a correct estimation of the QTL effects while most of the other methods underestimated them (Table 4). Only LASSO and the haplotype-based regression methods showed significant false positives, even if two suggestive false QTL were detected with the GBLUP. The significant false QTL were located in the 71.4-76.1 cM interval, which presents a slight LD with the two true QTL (Figure 3).

### Linked QTL in repulsion (Chr3)

Again, almost all methods identified the two QTL located at 5 and 15 cM (Figure 1C). More precisely, whereas the first QTL was correctly identified by all methods, the GBLUP [5] and single SNP approximated mixed model [13] missed the second QTL. The second QTL was also globally less precisely mapped than the first one (average distances: 1.19 cM versus 0.38 cM away from the real location). Interestingly, most of the methods correctly estimated the first QTL effect but largely underestimated the second effect. False positives were found with most of the methods (excluding BayesCπ and the two single SNP mixed models), with two major locations in the 26.7-28.9 cM and the 84.1-87.3 cM regions.

### Imprinted QTL (Chr4)

All genomic approaches except BayesCπ were able to detect the QTL (a suggestive signal was observed by Schurink et al. [8]) (Figure 1D). The local mixed model techniques did not detect this QTL, with the notable exception of GENMIX which in fact gave the most precise location of the QTL (only 0.5 cM away). It must be emphasized that none of the models underlying the methods assumed to possibility of such an imprinting effect. In addition, the accuracy of the QTL location was low, with an average distance to the true location of 3.4 cM. Only the GENMIX [7] method found the QTL at less than 0.5 cM from its real location. For this chromosome, many false positives were detected in two regions (55-62 cM and 90-98 cM), by methods that either did or did not map the imprinted QTL. While the power and accuracy differ between the methods for this imprinted QTL, none of the latter could correctly estimate its effect. All methods gave a very low effect for this quite large QTL.

### Interacting QTL (Chr5)

Finally, on chromosome 5, the first QTL was generally detected, with the exception of the GBLUP, BayesB, and BayesCπ in Zeng et al. [5] and of the approximated mixed model. Inversely, none of the methods was able to detect the second interacting QTL (Figure 1E). It must however be noted that a positive signal was obtained in the 91-92 cM interval by all of the mixed model approaches performed by Dashab et al. [7] and by the classical LASSO in Usai et al. [6] (this last result is less convincing as this method gave a very large number of false positives). This is surprising considering that a similar set of interacting QTL was simulated in the XIV^th ^QTLMAS dataset and was correctly mapped by all groups [14]. In addition, while one of the GBLUP tested by Zeng et al. [5] took epistasis into consideration, it did not map the second QTL. These results could be explained by the dominance hypothesis considered in the simulations, *i.e*. there is an effect at the first QTL only if there is the "1 1" genotype at the second QTL (Table 1 in [3]). Another group of false positives was also identified around 8.9 cM by the Bayes, LASSO and GENMIX methods. Again, none of the methods was able to estimate the QTL effect correctly.

## Conclusions

Considering all the results together, it is clear that the methods differ both in power and accuracy. The main cleavage is observed between additive and non-additive QTL detection. If most methods were able to detect the additive QTL located on chromosomes 1, 2 and 3, none of the methods mapped both interacting QTL on Chr5 and only 8 identified the imprinted QTL on Chr4. For this specific QTL, none of the regression-based methods (except the GENMIX approach) gave any results. If we overlook the LASSO method, which mapped very large regions with numerous false positives, the best results were obtained by Dashab et al. [7] with their BayesC and GENMIX methods (which identified 7 out of the 8 QTL). The BayesC method was particularly interesting as it showed only 2 false positives on all five chromosomes and it provided a good mapping precision, except for the imprinted QTL. If we consider the accuracy of the estimation of QTL effects, the two most efficient methods were GBLUP [5] and single SNP regression [7,13]. All Bayesian- and LASSO-based methods tended to underestimate the QTL effects. One interesting point to consider in the future would be to adapt the methods to more complex genetic situations since they represent a substantial part of the heritability of complex traits and they are not correctly allowed for in present methods, even those supposed to consider epistasis [5].

## List of abbreviations used

SNP: Single Nucleotide Polymorphism; QTL: Quantitative Trait Locus; GWAS: Genome Wise Association Studies; MCMC: Monte Carlo Markov Chain; LASSO: Least Absolute Shrinkage and Selection Operator; GBLUP: Genomic Best Linear Unbiased Prediction.

## Competing interests

The authors declare that they have no competing interests.

## Authors' contributions

OD and OF collected and processed the data files. OD and JME wrote the manuscript. All authors contributed to the ideas and methods, and read and approved the manuscript.
